# The Annual Dinner and the War

**Published:** 1915-05-29

**Authors:** 

**Affiliations:** President. 26 St. James's Place, S. W.


*To the Editor of* The Hospital.

### The Annual Dinner and the War.

*To the Editor of* The Hospital.

Sir,—In May of last year, when Mr. Justice Darling presided at the annual dinner in aid of the Royal Hospital for Incurables, Putney Heath, and when nobody imagined that war with Germany would be declared in the following August, I promised to preside at this year's annual dinner. The dinner is usually held in the early summer, and by means of it the sum of about £4,000 is raised annually. This year my colleagues on the Board of Management have decided that it will be better to abandon the dinner, in view of present national affairs.

I have just ascertained from the Secretary of the hospital that at this time last year the hospital had received £52,456 from the beginning of its financial year, namely, October 1. This year the hospital has received £18,109 only. Need I say any more than this? We have over nine hundred beneficiaries to whom we have pledged our word. I shall be very grateful if some of your many readers will send a contribution to this national charity. Owing to the abandonment of our annual dinner we are sure to lose still more income, unless the response to this brief appeal is very generous.—Yours faithfully,

Wolverton,

President.

26 St. James's Place, S.W.
May 25, 1915.

